# Evaluation of Membrane Ultrafiltration and Residual Chlorination as a Decentralized Water Treatment Strategy for Ten Rural Healthcare Facilities in Rwanda

**DOI:** 10.3390/ijerph121013602

**Published:** 2015-10-27

**Authors:** Alexandra Huttinger, Robert Dreibelbis, Kristin Roha, Fidel Ngabo, Felix Kayigamba, Leodomir Mfura, Christine Moe

**Affiliations:** 1 The Center for Global Safe Water, Sanitation and Hygiene at Emory University, 1518 Clifton Rd. NE, Atlanta, GA 30324, USA; E-Mails: ahuttin@emory.edu (A.H.); kmroha@emory.edu (K.R.); 2 School of Civil Engineering and Environmental Science, The University of Oklahoma, 455 West Lindsey, Dale Hall Tower 521, Norman, OK 73019, USA; E-Mail: rdreibe@ou.edu; 3 The Republic of Rwanda Ministry of Health Maternal and Child Health Unit; P.O. Box 84, Kigali, Rwanda; E-Mail: ngabog@yahoo.fr; 4 The Access Project Rwanda, P.O. Box 7393, Kigali, Rwanda; E-Mails: felix@theaccessproject.com (F.K.); leodomirm@theaccessproject.com (L.M.)

**Keywords:** low-income countries, chlorination, implementation, maintenance, membrane water treatment, operation, quality, sustainability, ultrafiltration

## Abstract

There is a critical need for safe water in healthcare facilities (HCF) in low-income countries. HCF rely on water supplies that may require additional on-site treatment, and need sustainable technologies that can deliver sufficient quantities of water. Water treatment systems (WTS) that utilize ultrafiltration membranes for water treatment can be a useful technology in low-income countries, but studies have not systematically examined the feasibility of this technology in low-income settings. We monitored 22 months of operation of 10 WTS, including pre-filtration, membrane ultrafiltration, and chlorine residual disinfection that were donated to and operated by rural HCF in Rwanda. The systems were fully operational for 74% of the observation period. The most frequent reasons for interruption were water shortage (8%) and failure of the chlorination mechanism (7%). When systems were operational, 98% of water samples collected from the HCF taps met World Health Organization (WHO) guidelines for microbiological water quality. Water quality deteriorated during treatment interruptions and when water was stored in containers. Sustained performance of the systems depended primarily on organizational factors: the ability of the HCF technician to perform routine servicing and repairs, and environmental factors: water and power availability and procurement of materials, including chlorine and replacement parts in Rwanda.

## 1. Introduction

A reliable supply of safe water is essential in health care facilities (HCF) for infection control and hygiene [1,2]. There is a fundamental need in low-income countries, particularly in Sub-Saharan Africa, to improve basic infrastructure for water, sanitation, and hygiene in HCF [3,4]. Insufficient water supply and substandard infrastructure (particularly sanitary facilities) have been documented as deterrents to seeking care, and contributors to staff absenteeism [4,5,6]. HCF have daily consumption-intensive needs for water including cleaning, laundry, and personal hygiene, and high quality water is particularly necessary for medical procedures and drinking needs [1]. A recent review by the World Health Organization (WHO) of 54 countries estimated that 42% of HCF in low-income countries do not have an improved water source within 500 m [7]. Among secondary and tertiary HCF that serve rural populations, water supply coverage is lower: HCF in rural, underserved areas in Kenya and Ethiopia had improved water supply coverage that was 22% and 72% lower, respectively, than in the capital. Where HCF do have connections to a piped water supply from an improved source (including protected wells and rainwater in rural areas), there is a risk of contamination because water flow is often intermittent and infrastructure is substandard [8,9,10]. Intermittent water supply, whether from a networked or non-networked source, necessitates storage of water in containers in order to have a reliable water supply, and this presents an additional risk of recontamination [11,12]. 

Decentralized on-site treatment, coupled with an adequate water supply, can provide high quality water in volumes suitable for small- to medium-scale applications, including HCF. Newer technologies allow high volume decentralized treatment systems (capacity ≥ 10,000 liters/day) to supply drinking water where centralized systems cannot reach populations or adequately meet demand [11,13,14]. Decentralized treatment using ultrafiltration (UF) technology for membrane water treatment is increasingly available and has growing potential for application in low-resource settings [13,14,15,16]. Published evaluations of decentralized systems using ultrafiltration membranes (0.01–0.10 μm membrane pore size, capable of high log-removal of protozoa and bacteria) for treatment of drinking water in low-resource settings largely consist of bench evaluations and trials that simulate a real-world application. Bench evaluations have demonstrated the UF membrane fouling rates, including the effects of oxidants and temperature [17,18,19,20]. In simulation trials, UF systems have been used to treat the same water sources used by the intended target populations, but the water treatment systems (WTS) were entirely managed by the research investigator [21,22]. These studies offer valuable evidence about the operational efficacy of WTS including evidence that the lifespan of membranes is highly dependent on fouling rates, constant and intermittent operation result in different membrane fouling patterns, and that natural organic matter in source water correlates strongly with fouling of membranes. Collectively, these bench evaluations and simulation trials demonstrate that UF can operate under less than ideal conditions; however, they have not addressed the feasibility and continued operation of advanced WTS or the necessary supporting infrastructure in a real-world low-income setting.

Only a small number of studies have assessed UF technology in a real-world setting, specifically decentralized WTS performance under actual *in situ* conditions and managed by local operators. In Mozambique and Ecuador, Arnal and colleagues described the technical performance of UF membrane WTS that supplied purified water to a hospital and a school. The Mozambique study evaluated design and installation of a hospital WTS and training of two local technicians for operations and maintenance, but operational constraints were not discussed [23]. The Ecuador study evaluated six months of the operation of a school WTS and demonstrated that it was feasible to integrate the WTS into existing local infrastructure to provide large volumes of purified water, but post-treatment chlorination was necessary to maintain water quality at the points of use [24]. Sima and colleagues examined existing supply models for purified water in Southeast Asia, noting that drinking water refill stations that use multi-stage treatment, including UF and chlorination, are a viable and growing market-based solution for safe drinking water provision [15]. The study discussed the appropriateness, profitability, and sustainability of these technologies for drinking water treatment in low-income settings, but did not provide details on operations and maintenance of the WTS, technical challenges, or factors contributing to success. Molelekwa described the application of a UF membrane WTS for a rural village water supply in South Africa, including technical and administrative engagement for the start-up of the pilot water treatment plant and training of one local technician; intermittent local supply of diesel fuel necessary to run the pump was identified as the principal constraint to water treatment system operation [25]. Each of these studies concluded that limited access to capital for start-up (for application outside of a donation model), weak supply chains for consumables, and lack of spare parts, tools and qualified technicians to perform maintenance and repairs, are significant barriers for the sustainable use of decentralized water treatment systems in low-income settings. The observations provided by Arnal and Molelekwa are limited to the operation and maintenance of one WTS at one site. This study describes system performance at ten sites and compares how site-to-site differences in WTS configuration, water and power availability, and operations and maintenance contributed to WTS functionality and water quality.

## 2. Experimental Section 

We conducted a prospective performance evaluation of WTS using membrane UF and residual chlorination that were installed in ten healthcare facilities in Rwanda in order to assess the feasibility of these systems to improve water quality in low-resource settings when placed in an institutional setting, and to proactively identify determinants of system sustainability. We collected a variety of data, including weekly operation logs, monthly water quality assessments, and maintenance and repair activity logs from the implementing organization, in order to identify the extent to which systems performed at capacity and to examine barriers to program success. We discuss our results within the framework of health service sustainability.

### 2.1. Study Setting 

This study was conducted among rural, secondary public health care facilities (classified as “health centers”) that are part of the Rwandan national health system. Health centers provide essential primary care services, including consultation, antenatal care, maternal care for normal deliveries, pharmaceuticals, family planning, pediatric care and nutrition, and laboratory diagnostics. The health centers were chosen to receive water purification systems donated by the General Electric (GE) Corporate Citizenship program Developing Health Globally™ based on the existing supporting infrastructure (water and power supply), maintenance staff, and existing oversight and support through district-level hospital affiliations. 

Health centers in the Northern, Eastern, and Western Provinces of Rwanda were targeted for participation. The Northern and Western Provinces have high population density and heavy rainfall, and improved water source coverage is lower than the rest of the country [26]. The Eastern Province experiences periods of drought, and the centralized water distribution system is recognized as needing major repair. These are also areas where Developing Health Globally maintains active health systems strengthening programs, thereby facilitating program delivery. 

### 2.2. Intervention 

#### 2.2.1. Inclusion Criteria and Site Selection

Inclusion criteria for health centers to receive the water purification system were: (1) year-round solar and/or grid power with outages lasting more that 24 h occurring less than once per month, (2) piped, well and/or rain water available on the plot, (3) water intended for drinking and medical purposes below WHO quality standards of for *E. coli*, total coliforms and residual chlorine, and (4) willingness of the health center director to receive the water purification system donation and participate in the research project. The Ministry of Health of Rwanda nominated 24 candidate health centers in the target areas, and 17 of these met the inclusion criteria. Two of the 17 opted out of the donation program because the management did not want to add a water purification system. The directors of these health centers believed the water quality, despite not meeting WHO guidelines, did not need to be improved using the WTS offered. Technical advisors visited the remaining 15 facilities and omitted two based on concerns about water infrastructure and seasonal water shortages. Three additional facilities were not included because of their relative isolation from population centers. A total of ten health centers were included in the intervention. 

#### 2.2.2. Water and Power Supply at Participating HCF

At the time of recruitment, three participating HCF had solar power and by the end of the observation period, all sites had grid power supplied by the national utility. All sites had some rainwater storage, ranging in volume from 13 to 100 m^3^. A forthcoming study by the authors characterizes the state of water and power supply, and infrastructure for water, sanitation and hygiene in the candidate and selected facilities with comparison to available information on the state of HCF in Rwanda and regionally in East Africa. 

#### 2.2.3. Water Purification Systems

The WTS had two core treatment processes: ultrafiltration (UF) and chlorination, and consisted of a 500 μm pre-filtration screen, two 0.02 μm UF units, and a hydraulically-driven pump to deliver dilute calcium hypochlorite solution post-filtration. The UF units were 55 m^2^ surface area of polyvinylidene fluoride (PVDF) hollow fiber membrane with outside-in flow path, flow range from 45 to 180 m^3^/day and trans-membrane pressure range from 0 to 40 psi [27]. The rejection rate for bacteria and viruses by the ultrafiltration unit was 99.9% [28]. The units were independently certified by NSF International for compliance with US and international standards. The estimated lifespan of the units for treating low turbidity water (<1 NTU), assuming proper maintenance, was over 20 years [29]. WTS were designed to have a peak output of 50,000 liters/day [30]. WTS were installed directly into the existing health center water distribution systems. Where possible, rainwater storage systems were integrated into WTS in order to reduce demand on metered water from local utilities. For this reason, electrically powered pumps were incorporated into the systems. The average cost of each water purification system was approximately 15,000 USD (equipment only) [30].

Technical advisors developed site-specific installation plans for each WTS that maximized integration of on-site rainwater catchment and minimized use of electricity while maintaining adequate water flow rates throughout the facility. The topography of each site and source water pressure influenced system configuration at the ten sites. There were five general configuration types: eight facilities received both pumps and pressure tanks, four post-treatment elevated storage tanks were constructed, at eight sites rainwater sources were integrated into the treatment system, and four underground post-treatment chambers were constructed to increase chlorine contact time before consumption. (See supplemental information for water purification system configuration diagrams.) Management of water supplies, including piped water, rainwater from on-site tanks, and water stored in holding tanks, was performed using manual valves in site-specific configurations. Bypasses were plumbed into all components of the system to allow isolation of components and to ensure water availability in the event of filtration system failure. Where possible, routine operation and maintenance tasks for the water purification system were manually operated (as opposed to automated) to reduce complexity. The pre-filter and ultrafiltration units had manual controls for cleaning procedures, and the chlorine dosing system was hydraulically-driven and did not require electrical power.

#### 2.2.4. Training and Start-Up

The WTS were donated and installed by GE’s corporate citizenship program Developing Health Globally™ in partnership with Assist International and the Government of Rwanda. WTS were installed and launched in two phases: March 2012 and December 2012. Health center maintenance staff were identified as the primary operators of the WTS and were trained on daily and weekly operations and maintenance through demonstrations and visual aids (poster diagrams in the local language in the MF system buildings). Trainings delivered to system operators focused on routine operations and maintenance, including: daily backwashing of the UF units, weekly cleaning of the pre-filter, weekly preparation of chlorine solution, and valve configuration for optimizing water use and storage. 

Priorities during this start-up phase were: (1) ensuring sound plumbing and electrical work to integrate the water purification systems into the existing health center infrastructure, and (2) providing on-going training to health center staff for WTS routine operations and maintenance. Preventative maintenance beyond daily and weekly tasks performed by the on-site operators, such as routine servicing of chlorine dosing systems did not occur during the observation period. 

Following the initial installation and commissioning of the WTS and training health center staff in routine operation and maintenance, the WTS were operated and managed by health center staff with continued support from the implementing organization through a service contract with local contractors. A field coordinator: (1) provided oversight for plumbing and electrical work executed by local contractors, (2) coordinated response to repair needs, and (3) delivered on-going training to the HCF technical staff for routine operations and maintenance. No training on system component servicing (such as pump or chlorine dosing systems) was provided to system operators. The implementing organization determined that the level of complexity of those operations was beyond the technical capacity of health center personnel, and necessary tools were not available at any health center. 

### 2.3. Program Monitoring 

#### 2.3.1. Data Collection and Monitoring

Routine data collection started in March 2012 and continued through December 2013. Performance and operation were assessed through two primary data collection activities: 

*Weekly WTS assessments*: Once per week, the field coordinator visited each health center to inspect the water purification system and report on operations, routine maintenance, and any abnormalities in system functions. Additionally, daily records maintained by system operators were checked for completeness. Daily records included water meter readings and pre- and post- backwashing pressure before and after the ultrafiltration unit. These reports were compiled and digitized by trained field investigators on a weekly basis. 

*Monthly Facility surveys*: Monthly facility surveys were performed during unannounced site visits by trained field investigators working independently from the implementation team. Regardless of purification system operation, field investigators observed water availability and collected water samples immediately following the WTS and from points of use in surgery, maternity, male and female wards, and pharmacy. For additional buildings outside of those predefined services, such as administration, laboratory and voluntary counseling and testing, water availability was observed and a sample was collected from one point of use per building. Points of use in all services were sinks with faucets and improved storage containers. During water interruptions, alternative sources included rain tanks and water from containers filled off-site. If the point of use was a container, water was only sampled if it was reported that the water was used for drinking. Duplicate samples were collected from each sampling point.

Performance data from the WTS system weekly assessments and monthly facility surveys were combined in event logs of systems operation. The log was supplemented with information about repairs performed by the implementation partner and local contractors from the day of installation through December 2013 for each facility. 

#### 2.3.2. Data Analysis

The WTS event logs were analyzed in Microsoft Excel (Redmond, WA, USA). These data were used to classify each site-specific day of operation as either fully operational or experiencing service interruptions with an identified cause. For a system to be classified as fully operational, three criteria were assessed: (1) there was piped water and electricity available on site; (2) the filtration system and associated components were working as intended; and (3) the filtration system was used as intended. Service interruptions included any event in which these criteria were not met. Service interruptions were further classified into *water interruptions* in which water was not available, or *treatment interruptions* in which some part of the treatment process, such as the chlorine dosing system, was compromised. We report system performance as the total number of days that the WTS were fully operational out of the total number of days of observation. For reported repair needs, the number of days that passed between reporting the problem and repair were used to calculate time to resolution. For key components that failed during the observation period, we calculated time to failure as the number of days from installation until the time the particular component failed. Mean time to resolution and time to failure were calculated for comparable issues and components of the WTS across the ten sites.

Water samples were collected immediately following the treatment system (chlorinated UF permeate) and from points of use in each service of the health centers. Water samples were placed on ice and processed within 3 h to assess levels of chlorine residual and turbidity, and within 12 h of collection to assess concentrations of *E. coli* and total coliforms. Physio-chemical testing was performed using portable digital meters (Hach Co., Loveland, CO, USA) and the DPD technique for chlorine residual detection. Water samples were tested for total coliforms and *E. coli* using the Quanti-Tray method and Colilert growth medium (IDEXX Laboratories, Inc., Westbrook, ME, USA). Quanti-Tray method estimates the most probable number (MPN) of colony forming units of microorganisms. The lower and upper detection limits were <1 and 2419.6 MPN/100 mL. Frequencies or means of key descriptive variables were calculated. 

Water quality measures were compared to system performance data in order to examine water quality by WTS status at the time of sample collection (fully operational, treatment interruption, or water interruption). Water quality measures were also stratified by sampling point (water sampled directly from a tap or from a storage container). Unadjusted odds ratios were calculated for presence of total coliforms/*E. coli* in 100 mL samples by WTS status and sampling point. Water quality measures for samples of chlorinated UF permeate collected immediately following the treatment system were analyzed separately. All water quality data were analyzed with SAS v9.3 (SAS Institute, Cary, NC, USA).

#### 2.3.3. Ethics

The study was reviewed and approved by the Institutional Review Board at Emory University (No. IRB00053040, as amended), and the Rwanda National Ethics Committee (No. 646/RNEC/2014).

## 3. Results and Discussion

### 3.1. Water Purification System Performance 

#### 3.1.1. Operations and Maintenance

**Figure 1:**
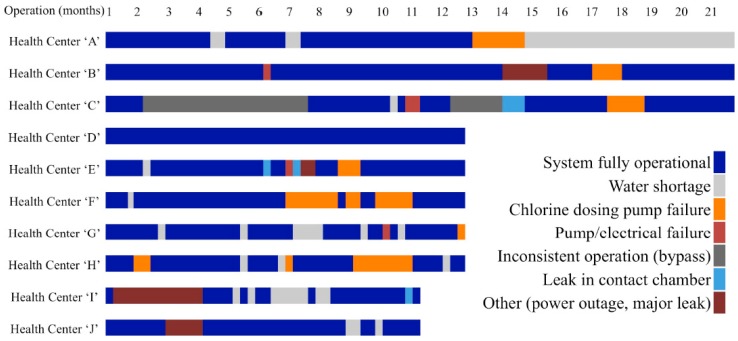
Event log timeline of water treatment system service interruptions and causes at ten health centers in rural Rwanda, March 2012–December 2014.

Operations, maintenance, service, and repairs were monitored from the day of installation through December 2013, with a mean observation period of 439 days (range: 320–621 days per site). Overall, the WTS were fully operational for 74% of the observation period. WTS at five sites were fully operational for >80% of the observation period, four were fully operational for 59%–74% of the observation period, and one was operational for <50% of the observation period (range: 40%–100% per site) (Figure 1). This corresponds to a total of 256 days of service interruption per 1000 days of observation. Of the 1130 days of service interruption during the observation period, 36% were treatment interruptions and 64% were water interruptions that resulted in no provision of piped water at the health centers (Table 1).

**Table 1:** Causes and duration of water treatment system service interruptions at ten health centers in rural Rwanda, March 2012–December 2014.

	Treatment Interruption *		Water Interruption **			
**Reason for service interruption**	User bypass	Chlorine dosing pump failure	Water shortage	Pump/electric failure	Underground contact chamber leak	Other ^1^
**Number of events**	2	12	22	4	5	4
**Number of sites at which events occurred**	1	7	8	4	4	4
**Days of interruption (proportion of total observed interruption period)**	91 (0.08)	315 (0.28)	367 (0.32)	218 (0.19)	75 (0.07)	64 (0.06)
**Mean time to failure in days (range) ^2^**	N/A	330 (61–542)	N/A	161 (2–390)	300 (171–427)	N/A
**Mean time for repairs to be completed in days (range) ^2^**	N/A	24 (1–37)	N/A	55 (15–125)	18 (4–53)	N/A

* Treatment interruption indicates periods when the mechanisms for ensuring safe water at the point of were compromised. ** Water interruption indicates periods when the piped water supply was not available. ^1^ Reasons for other service interruptions: 3 power outages, 1 major leak. ^2^ Time to failure and time for repairs to be completed were not recorded for user bypass, water shortage or other interruptions because these incidents were not attributed to events caused by the infrastructure modification made by the program in order to integrate the WTS into health center piped water systems.

#### 3.1.2. Treatment Interruptions

**Figure 2:**
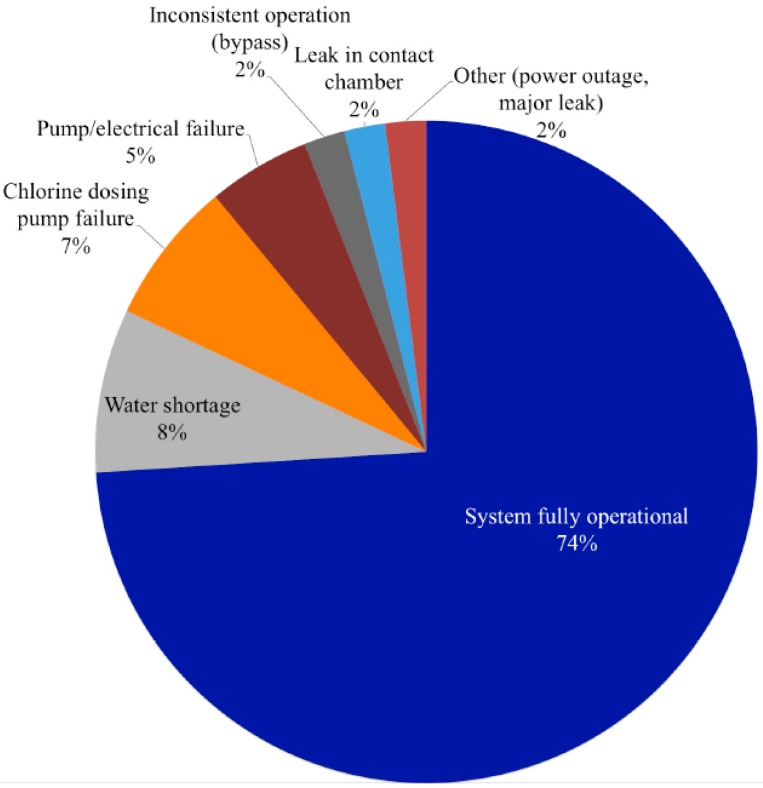
Functionality of water treatment systems at ten health centers in rural Rwanda, March 2012–December 2014.

Treatment interruptions occurred for 9% of the total observation period and accounted for 36% of service interruptions overall. The most common cause of treatment interruption was failure of the chlorine dosing system, which occurred 12 times at seven sites (Figure 2). The 12 observed events were the same type of problem: loss of suction due to strain on, and abrasion to, gaskets. The average time to failure for chlorine dosing systems was 330 days (range: 61–542 days). Treatment interruptions due to chlorine dosing system failures accounted for 70 days per 1000 days of observation across all sites. 

During the initial start-up period, short instances (1–3 days) of user bypass were observed at half of the sites. Reasons for bypass included failure to turn system on after a water or power shortage and health center visitors tampering with valves. Persistent and intentional bypassing of the water purification system only occurred at one site (site “C”), accounting for 8% of days of service interruption (<2% of the total observation period) (Figure 2). At this site, reasons offered by health center staff for bypassing the water purification system included greater expenditure on water following system start-up, wastage of water during the daily UF unit backwash cleaning procedure, and leaks within the piped water infrastructure within the health center. Additionally, the way the WTS was integrated into the piped water infrastructure involved 11 manual valves for daily operations, as compared to the simplest configuration (site “D”), which involved four valves (supplemental Figures S1–S5 depict, from most to least complex, the five configurations of WTS that were applied). 

#### 3.1.3. Water Interruptions 

The most frequent and widespread reason for water interruption was water shortage at the source, usually a municipal distribution system supply. Water shortage accounted for 32% of the total service interruptions (9% of the total observation period) (Figure 1). Only two sites did not experience a water shortage during the observation period, and the average duration of water shortage events was 15 days (median: 10 days, range: 1–70 days) (Table 1). 

Pump or electrical failures resulted in four water provision interruptions, each occurring at a separate site (Figure 2). These included: failure of an existing pump, failure of a newly installed pump, failure of a newly installed electrical switch, and failure of a newly installed solar energy system due to storm damage. These failures occurred at random and did not indicate a specific design or component weakness. Pump or electrical failures accounted for 19% of all water interruptions (5% of total observation period) (Figure 1). Problems with chlorine contact chambers, installed in four health facilities, accounted for 7% of all water service interruptions. Leaks at joints of different width or leaks at elbows in the contact chambers occurred at three of the four sites with this design feature; these were significant leaks that necessitated shut off of the water main. These problems indicated a weakness in the construction executed on-site by local contractors. Time to failure for chlorine contact chambers ranged from 180 to 425 days (Table 1). 

#### 3.1.4. Time to Resolve System Interruptions

The duration of all system interruptions, excluding water shortages, power outages, and user bypass, was dependent on the response time of local contractors hired by the implementing organization. For all technical problems, including chlorine dosing system failures, electrical or pump failures, or chlorine contact chamber leaks, the average time to resolve the problem was 30 days. Time to resolve was shortest for leaks in contact chambers (average: 18 days) and chlorine dosing system failures (average: 24 days). Average time to resolve pump or electrical failures was 55 days (range: 15–125 days) (Table 1).

In addition to technical problems that caused service interruptions, minor events such as small leaks associated with the WTS were also reported. During the observation period, there were 30 minor events and the average time to resolve was 27 days. 

### 3.2. Water Quality 

Because of changes in water availability at specific sampling points in each health center between rounds of data collection, the number of water samples collected varied from month to month. In the 12 months of water quality monitoring, a total of 592 water samples were collected: 446 samples when the systems were fully operational, 96 samples during treatment interruptions, and 50 samples during water interruptions. Forty-seven samples were collected directly following the MF systems: 40 samples when the systems were fully operational, and seven samples during quality interruptions. 

Water samples were not collected from the primary piped water and rain water supplies (pre-treatment) during the monthly water quality observations. However, source water from rain tanks was collected and tested during the pre-intervention baseline assessment: total coliforms were detected in 91% of samples and *E. coli* in 17% of samples, the mean turbidity was 2.10 NTU (range: 0.17–7.31). All source water samples, including the piped water supplies, had <0.1 mg/L of residual free chlorine [31].

#### 3.2.1. Quality of Water in Samples of Chlorinated UF Permeate Collected Immediately Following the WTS

When the WTS were fully operational, concentrations of total coliforms and *E. coli* were less than one MPN in all 100 mL samples (N = 40). The mean free chlorine residual was 0.43 mg/L, but the median free chlorine residual was 0.02 mg/L, which was the lower limit of detection of the digital colorimeter used to measure chlorine residual. Only 11 of 40 samples (28%) had free chlorine residual that met the World Health Organization (WHO) guideline of ≤0.2 mg/L. (Results are presented in supplemental information Table S1). During treatment interruptions, >1 MPN/100 ml total coliforms were found in one of six samples, and no samples had >1 MPN/100 ml *E. coli*. The median free chlorine residual was 0.02 mg/L (results are presented in supplemental information). The WTS were not in operation during water interruptions and thus chlorinated UF permeate samples were not available.

#### 3.2.2. Quality of Water at Points of Use

When the WTS were fully operational, 397 (89%) of water samples collected from points of use within the health centers—including water sampled from storage containers—met the WHO guideline for drinking water quality of <1 MPN total coliforms per 100 mL, and 432 (97%) of samples met WHO guideline of <1 MPN *E. coli* per 100 mL. The mean free chlorine residual was 0.09 mg/L (range: 0–1.9). During treatment interruptions, when piped water was flowing but the WTS was not fully operational, 79 (82%) of the 96 samples met WHO guidelines for total coliforms, and 93 (97%) met WHO guidelines for *E. coli*. The mean free chlorine residual was 0.02 mg/L (range: 0–0.17) (Table 2).

Of the 369 tap water samples collected when the WTS were fully operational, 344 (94%) of samples met the WHO guideline for total coliforms, 363 samples (98%) met the WHO guideline for *E. coli*, and the mean free chlorine residual was 0.1 mg/L (range: 0–2.20). Eighty-three samples were collected from taps during treatment interruptions, 70 (84%) of samples met the WHO guideline for total coliforms, 81 (98%) met the WHO guideline for *E. coli*, and the mean free chlorine residual was 0.02 mg/L (range: 0–0.17) (Table 3). During treatment interruptions, samples collected from taps were 2.7 times more likely to have one or more total coliforms MPN per 100 mL (OR: 2.7, 95% CI: 1.3–5.5) compared to tap samples collected when the WTS were fully operational. (Table 3), and there was no significant difference (*p* = 0.6) in the proportion of tap samples with >1 MPN *E. coli*/100 mL when the WTS were operational *vs*. during treatment interruptions. 

**Table 2:** Quality of water from samples collected at points of use when water treatment systems were fully operational, during treatment interruptions, and water interruptions.

	WTS Fully Operational n (%)	Treatment Interruption * n (%)	Water Interruption ** n (%)
Number of samples	446	96	50
***E. coli* (MPN** ^†^**/100 mL)**			
<1	432 (96.9)	93 (96.9)	39 (78.0)
1–10	11 (2.5)	1 (1.0)	2 (4.0)
>10	3 (0.6)	2 (1.2)	9 (18.0)
**Total Coliforms (MPN** ^†^**/100 mL)**			
<1	397 (89.2)	79 (82.3)	31 (62.0)
1–10	26 (5.8)	7 (7.3)	3 (6.0)
>10	22 (5.0)	10 (10.4)	16 (32.0)
Number of samples	440	84	47
**Free chlorine residual (mg/L)** ^††^			
Mean	0.12	0.02	0.03
Median	0.02	0.02	0.02
Range	<0.02–2.20	<0.02–0.17	<0.02–0.35
**Total chlorine residual (mg/L)** ^††^			
Mean	0.18	0.06	0.08
Median	0.06	0.04	0.04
Range	<0.02–2.20	<0.02–0.26	<0.02–0.48
**Turbidity (NTU)**			
Mean	1.13	1.22	3.36
Median	0.70	0.74	1.34
Range	0.02–26.63	0.05–6.92	0.39–49.61

* Treatment interruption indicates periods when the mechanisms for ensuring safe water at the point of use, such the chlorine dosing mechanism, were compromised. ** Water interruption indicates periods when the piped water supply was not available, such as during interruptions in the piped water supply and during pump failures; see Table 1 for causes and duration of events. ^†^ Most Probable Number. ^††^ Limits of detection for free and total chlorine residual were 0.02 to 2.20 mg/L.

**Table 3:** Quality of water from samples collected from taps and storage containers when water treatment systems were fully operational and during treatment interruptions.

	WTS Fully Operational		Treatment Interruption *	
	TAPS n (%)	Containers n (%)	Taps n (%)	Containers n (%)
Number of samples	369	77	83	13
*E. coli* (MPN **/100 mL)				
<1	363 (98.4)	69 (89.6)	81 (97.6)	12 (92.3)
1–10	6 (1.6)	5 (6.5)	1 (1.2)	0 (0)
>10	0 (0.0)	3 (3.9)	1 (1.2)	1 (7.69)
Total Coliforms (MPN **/100 mL)				
<1	344 (93.5) ^†^	53 (68.8)	70 (84.3) ^†^	9 (69.2)
1–10	16 (4.4)	10 (13.0)	6 (7.23)	1 (7.69)
>10	8 (2.1)	14 (18.2)	7 (8.43)	3 (23.8)
Number of samples	364	76	83	13
**Free chlorine residual (mg/L)** ^††^				
Mean	0.13	0.11	0.02	<0.02
Median	0.02	0.02	<0.02	<0.02
Range	<0.02–2.20	<0.02–2.20	<0.02–0.17	<0.02–0.04
**Total chlorine residual (mg/L)** ^††^				
Mean	0.19	0.15	0.06	0.02
Median	0.07	0.04	0.04	0.02
Range	<0.02–2.20	<0.02–2.20	<0.02–0.26	<0.02–0.09
**Turbidity (NTU)**				
Mean	1.09	1.30	1.17	1.49
Median	0.71	0.62	0.74	0.74
Range	0.02–20.07	0.12–26.6	0.05–6.92	0.12–5.76

* Treatment interruption indicates periods when the mechanisms for ensuring safe water at the point of use, such the chlorine dosing mechanism, were compromised. ** Most Probable Number. ^†^ During treatment interruptions, samples collected from taps were 2.66 times more likely to have one or more total coliforms MPN per 100 mL (OR: 2.7, 95% CI: 1.3–5.5) compared to tap samples collected when the MF systems were fully operational. ^††^ Limits of detection for free and total chlorine residual were 0.02 to 2.20 mg/L.

#### 3.2.3. Quality of Water in Storage Containers

Because of the realities of intermittent water supply at health centers, incomplete coverage of the piped water network in all parts of the health center, and delays in fixing broken taps, post-treatment storage of water, in jerry cans and improved storage containers (defined as having a narrow mouth and a spigot for water access), was regularly observed at all sites. When WTS were fully operational, 82% of water samples were collected from taps and 18% were collected from storage containers. Among the 77 water samples collected from containers when the treatment systems were fully operational, 53 (69%) met the WHO guideline for total coliforms, 69 (90%) met the WHO guideline for *E. coli,* and the mean free chlorine residual was 0.11 mg/L (range: 0–2.20) (Table 3). When the WTS were fully operational, water samples collected from containers were 6.5 times less likely to meet the WHO guideline for total coliforms than samples collected from taps (OR: 6.49, 95% CI: 3.43–12.25), and were 7 times less likely to meet the WHO guideline for *E. coli* (OR: 7.01, 95% CI: 2.36–20.85) (Table 4). The mean concentration of free chlorine in water samples collected from containers was 0.11 mg/L (range: 0–2.20), marginally lower than the mean concentration in samples collected from taps (Table 3).

During treatment interruptions, 13 samples were collected from containers; 9 (69%) of these samples met the WHO guideline for total coliforms, 12 (92%) met the WHO guideline for *E. coli* and the mean free chlorine residual was 0.01 mg/L (range: 0–0.04) (Table 3). There was not a significant difference between the proportion of water samples from containers with >1 MPN/100 mL total coliforms or *E. coli* collected during treatment interruptions and when the WTS were fully functional. Considering all the water samples (from taps and containers), the odds of a water sample collected during treatment interruption meeting the WHO guideline for total coliforms or *E. coli* were not significantly different compared to when the WTS systems were fully functional (total coliforms OR: 1.00, 95% CI: 0.28–3.53; *E. coli* OR: 1.78, 95% CI: 0.97–3.25) (Table 4). 

Fifty water samples were collected during water interruptions (when piped water was not available). The majority of these samples were collected from alternative water sources: rain water tanks (no treatment) or containers that were filled off-site and may have been treated with a disinfectant. Thirty-one samples (62%) met the WHO guideline for total coliforms, 79 (78%) met the WHO guideline for *E. coli,* and mean free chlorine residual was 0.03 mg/L (range: 0–0.35) (Table 2). Considering all water samples from taps, containers, and alternative sources during water interruptions, the odds of a water sample meeting the WHO guideline for total coliforms during water interruptions was over five times lower compared to when the WTS were fully functional (OR: 5.1, 95% CI: 2.66–9.66), and the odds of a water sample meeting the WHO guideline for *E. coli* during water interruptions was over 8 times lower compared to when the WTS were fully functional (OR: 8.70, 95% CI: 3.70–20.47) (Table 5).

**Table 4:** Proportions and odds ratios of water samples with total coliforms and *E. coli* collected from taps and containers when water treatment systems were fully operational.

	≥1 total coliform MPN ^†^/100 mL		≥ 1 *E. coli* MPN ^†^/100 mL	
Point of Use Type	n (%)	OR (95% CI)	n (%)	OR (95% CI)
**Tap**	24 (6.5)	ref	6 (1.6)	ref
Container *	24 (31.2)	6.49 (3.43–12.25)	8 (10.4)	7.01 (2.36–20.85)

* Jerry can or improved water storage container. ^†^ Most Probable Number (MPN).

**Table 5:** Proportions and odds ratios of water samples with total coliforms and *E. coli* collected when water treatment systems were fully operational, during water quality interruptions and during water provision interruptions *.

	≥ 1 total coliform MPN ^†^ /100 mL		≥ 1 *E. coli* MPN ^†^/100 mL	
System Status	n (%)	OR (95% CI)	n (%)	OR (95% CI)
**WTS Fully Operational**	48 (11)	ref	14 (3)	ref
Treatment Interruption **	17 (18)	1.78 (0.97–3.25)	3 (3)	1.00 (0.28–3.53)
Water Interruption ***	19 (38)	5.07 (2.66–9.66)	11 (22)	8.70 (3.70–20.46)

* Analysis inclusive of water samples collected from taps and containers when WTS were fully operational and during treatment interruptions, and samples collected from rain tanks and containers filled off-site during water interruptions. ** Treatment interruption indicates periods when the mechanisms for ensuring safe water at the point of use, such the chlorine dosing mechanism, were compromised. *** Water interruption indicates periods when the piped water supply was not available, such as during interruptions in the piped water supply and during pump failures; see Table 1 for causes and duration of events. ^†^ Most Probable Number.

### 3.3. Discussion 

#### 3.3.1. Determinants of MF System Performance

**Figure 3:**
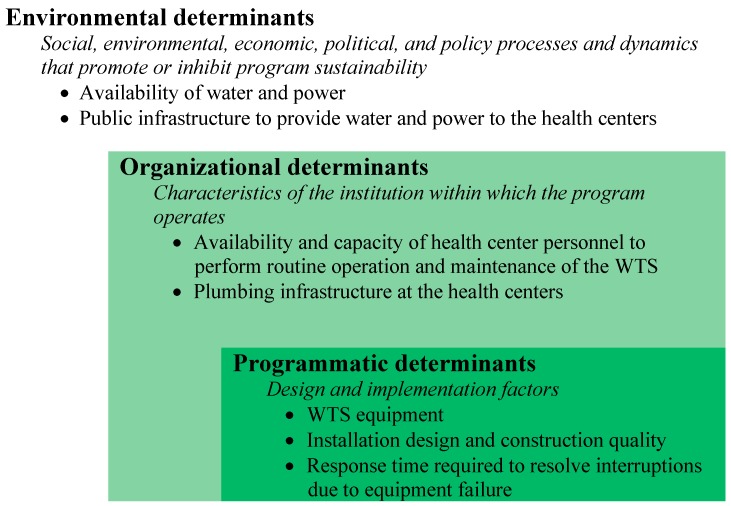
Determinants of water treatment system performance derived from sustainable health services delivery framework *. (* Adapted from Sarriot *et al.* 2004 [34] and Schreier *et al.* 2011 [32].)

Based on 22 months of data collection, providing an average of 439 days per site, it is clear that this WTS is a feasible on-site water treatment option for some healthcare facilities in low-income settings. Water and power were supplied by public utilities to the health centers for 90% of the observation period. When water and power were available, the treatment systems functioned as intended 82% of the time. The impact of integration of rainwater into the WTS and the impact of solar *versus* grid power, are complex and beyond the scope of this paper. Continuous operation of the WTS depended on a number of factors that are also fundamentally important to sustained use. Research on the sustainable delivery of health services provides a useful framework for understanding the feasibility and potential sustainability of WTS in healthcare facilities in low-income countries [32,33,34,35]. In a review examining the sustainability of health intervention programs both in the US and abroad, Shediak-Rizkallah and Bone (1998) frame the need for conceptual and operational definitions of sustainability; they identify “*three major categories that determine program sustainability: project design and implementation factors, organizational factors, and environmental factors”* [21]. In the context of this study, the aspects of project design and implementation that influence water purification system technical performance are: the equipment, installation design and construction quality, and the response time required to resolve interruptions due to equipment failure. Organizational determinants of the water purification system performance were the availability and capacity of health center personnel to perform routine operation and maintenance of the WTS, and the plumbing infrastructure at the health centers. Environmental determinants of performance included the availability of water and power, and the public infrastructure that provided water and power to the health centers (Figure 3). 

#### 3.3.2. Factors Associated with Successful Operation of MF Systems

Health center personnel adhered to routine operations and maintenance tasks, and maintained the water infrastructure at the health centers to the best of their ability. In contrast to community-based WTS, these health centers provided a ready pool of potential operators—the health center maintenance staff—who were technically proficient, equipped to follow the instructions for routine maintenance and operation of the system, and able to take on the additional responsibilities for maintaining systems. The daily and weekly operation and maintenance tasks did not require high levels of technical specialization and only required about 15 min per day. Deficits in the organizational determinants of performance accounted for less than one fifth of the total observed service interruptions. 

Similar to studies by Arnal in Mozambique and Ecuador, and Molelekwa in South Africa, we found that the membrane UF component of the WTS demonstrated robust performance, and trained operators were able to use and maintain them. In a review of decentralized systems for water purification, Peter-Varbanets *et al.* (2009) [13] identify that ultrafiltration systems had reliable performance, were easy to use and had simple maintenance procedures. There were no service interruptions due to the ultrafiltration component of the water treatment systems. However, the overall performance was limited by other more labor-intensive demands of the system hardware, specifically the service and repair needs of chlorine dosing systems.

Simplicity of design and quality of construction facilitated successful operation of the WTS. In some sites, we observed that poor basic construction practices for trenching, laying, and joining pipes resulted in early repair needs that required substantial investment from the implementation partner to resolve. Site “D” (Supplemental Figure S5) had the least complex design and was the only site at which we observed uninterrupted operation of the WTS, whereas, site “C” (Supplemental Figure S1), where intermittent operation was observed, had the most complex configuration. 

Moreover, construction quality, as an aspect of the project implementation, was an important determinant of system performance and durability: sites with less plumbing, *i.e.*, without chlorine contact chambers and multiple rain tanks, had fewer treatment interruptions due to leaks and pipe breaks. 

#### 3.3.3. Factors Associated with Interruptions in Water Provision and Water Treatment

Interruptions in water provision and water treatment occurred for 26% of the observation period. Seventy percent of the observed time when the systems were not operating was due to programmatic determinants: frequent chlorine dosing system failures and the long time to resolve service interruptions were the major contributing factors. Time to resolution varied by the complexity of the hardware failure and the availability of a trained service technician from the implementing organization. All of the health centers were within three hours travel time to the capital city, and spare parts were stocked in country by the implementing partner. 

Environmental factors accounted for one third of the days of service interruption, largely due to water shortages and, to a much lesser extent, power outages. Notably, the health centers selected for this intervention had more robust access to water and electricity than other health centers in Rwanda and the majority of health centers and communities in Sub-Saharan Africa [3,36]. In a similar program in six hospitals in Ghana where GE Foundation installed WTS in 2006, the predominant reason for interruption in water treatment was lack of water supply (unpublished data).

#### 3.3.4 Factors Affecting Water Quality at Point of Use

Overall, microbiological water quality in the health centers was good, but free chlorine residual was consistently below the WHO guideline intended for residual protection at points of use. The most common cause of service interruption attributable to internal factors in the WTS (excluding external factors of water shortage, power outage, and user bypass) was failure of the chlorine dosing system, and even when the WTS were fully functional, the median free chlorine residual in point-of-use water samples was negligible. Chlorine was provided to the health centers by the implementing organization in bulk quantities, and the system operators consistently and correctly prepared chlorine solution. However, the chlorine dosing systems did not deliver consistent water treatment: less than one third of water samples collected immediately following the WTS had free chlorine residual that met the WHO guideline for point-of-use water quality (Supplemental Table S1). The absence of routine servicing of chlorine dosing systems during the observation period, and the extent of failure of the chlorine dosing systems themselves, were driving programmatic determinants of the overall WTS performance during the observation period. 

Due to limited piped water infrastructure and frequent interruptions in water supply, health centers used point-of-use water storage containers to provide water for hand washing and drinking. When WTS were fully functional, one fifth of the water samples collected at the point-of-use came from storage containers. While storage containers provided an immediate solution for the need to provide adequate quantities of water, storing water in containers presents a risk of re-contamination and biofilm formation that is well documented in household water quality literature [11]. Adequate levels of residual disinfectant are essential for maintaining water quality in the piped water network and where containers are used. We observed significant deterioration of water quality in samples taken from containers *versus* taps. This is similar to findings from evaluations of WTS in Ecuador and Mozambique where post-treatment disinfection using chlorination was necessary to achieve recommended drinking water quality at the point of distribution [23,24]. 

#### 3.3.5. Study Strengths and Limitations

This assessment is one of the first prospective performance evaluations of a WTS using membrane UF in a low-income country. We systematically monitored the technical operation and performance of the WTS and objectively evaluated the reasons for failure. We examined not only the technology and the context in which these systems were operated, but also how the implementation of the program influenced the performance and overall outcomes. We evaluated water quality at the WTS and at points of use within the health centers to examine the effectiveness of the treatment technology and post-treatment changes in water quality due to the condition of infrastructure and hygiene practices in these health care facilities.

Communication between partners allowed for evidence-based decisions to improve the implementation of this project. The need to train HCF staff in non-complex routine maintenance and servicing, and to train Rwanda Ministry of Health technicians to perform complex servicing and repairs was identified, and a nine-month training program was initiated in December 2014. The necessity for a more robust chlorine dosing system was identified and systems were installed in the final months of the observation period.

There are specific limitations to this study: the purposive selection of a small number of health centers able to meet the criteria for participation in the intervention was not representative of health centers across Rwanda or healthcare facilities in Sub-Saharan Africa. However, we recognize that this technology requires the appropriate niche of environmental and organizational determinants for the system to add value through reliably providing large volumes of purified water. The timing of this research did not allow us to follow the performance of the WTS after the donor-sponsored implementation and monitoring period. Our observations are therefore limited to the feasibility and performance of the WTS during a time of intense oversight from both the research and the implementing organizations. This study did not include an exploration of the costs of WTS operation, maintenance, servicing and repair. Evaluation of the life cycle costs of water supply and water purification systems are valuable for considering organizational and environmental factors that affect sustainability, including access to operation and maintenance funds, supply chain for spare parts, and the need to train and re-train operators and technicians to overcome staff turn-over [37,38]. Offsetting the costs of maintenance and operation of these systems is currently being explored through the integration of public kiosks that sell the treated drinking water at the health center to the populations within the health center catchment area, a model commonly employed in community-based settings. A forthcoming study by the authors examines the impact of these kiosks on the financial sustainability of the WTS for the health centers and on community drinking water practices and quality.

## 4. Conclusions 

WTS utilizing membrane UF are a feasible on-site water treatment method for health care facilities and perhaps other institutions in low-income countries. These systems are capable of producing large volumes of high quality water; however, their application is limited to areas with robust access to water supply, and in most instances, electrical power. In settings where post-collection storage is common or even necessary, residual disinfection is essential for maintaining water quality. The routine operation and maintenance activities for the membrane UF component of the WTS studied did not require technical expertise, nor did it demand substantial time investment from the operator, whereas, the chlorination component frequently required maintenance and repairs that were beyond the capacity of the operator. We observed some deterioration of microbiological water quality during treatment interruptions and when water was stored in containers. We conclude that the low chlorine residual was insufficient to protect the water from post-treatment contamination in the health center plumbing network and in storage containers. 

This intervention focused on health centers in rural areas of Rwanda, yet the implications of our research inform the application of WTS in all settings where there are limitations in infrastructure, resources, capital, and/or human capacity. Our findings underscore the important role of programmatic factors, organizational determinants, and environmental constraints in the viability of WTS for low-resource settings. Communication between partners allowed for evidence from Rwanda HCF to be applied to improve the WTS components and the implementation of the program. This accelerated learning has been applied to similar programs delivered in low-income settings in Africa, Asia and Latin America.

In this intervention, internal health care facility personnel were responsible for WTS operation and demonstrated the capacity and appropriate oversight to ensure daily activities were performed. However, the external implementing organization identified and resolved all system repair needs, supplied consumable materials for chlorine disinfection, and assessed chlorine residual and microbiological water quality. In order for sustained operation of WTS beyond the end of the donor-sponsored program, supplies procurement, and system maintenance and repair needs that exceed the capacity of operators will need to be transferred to the regional and national health system and integrated into the management structures of those organizations. The implementation of WTS in other institutional or community-based settings, particularly where water flows in complex, piped networks or is stored in containers, must consider the person-time for routine operations, availability of technicians qualified to perform routine maintenance and repairs, and supply chains for replacement parts and chemicals for residual disinfection. Decisions to invest in WTS *versus* other interventions to improve quality of care and infection control—such as improving quantity and availability of water, improving sanitary facilities or solutions for hand washing at healthcare facilities—should be carefully considered as all of these factors are critical for safe heath service delivery.

## Supplementary Files

Supplementary File 1
