# Supplementary material for: Evaluation of Membrane Ultrafiltration and Residual Chlorination as a Decentralized Water Treatment Strategy for Ten Rural Healthcare Facilities in Rwanda
Source: Int J Environ Res Public Health. 2015 Oct 27;12(10):13602–23. doi: 10.3390/ijerph121013602 (PMC4627051; doi:10.3390/ijerph121013602)
Supplement: Supplementary File 1 [file ijerph-12-13602-s001.pdf]

Supplementary Information

Evaluation of Membrane Ultrafiltration and Residual Chlorination as a Decentralized Water Treatment Strategy for Ten Rural Healthcare Facilities in Rwanda

---

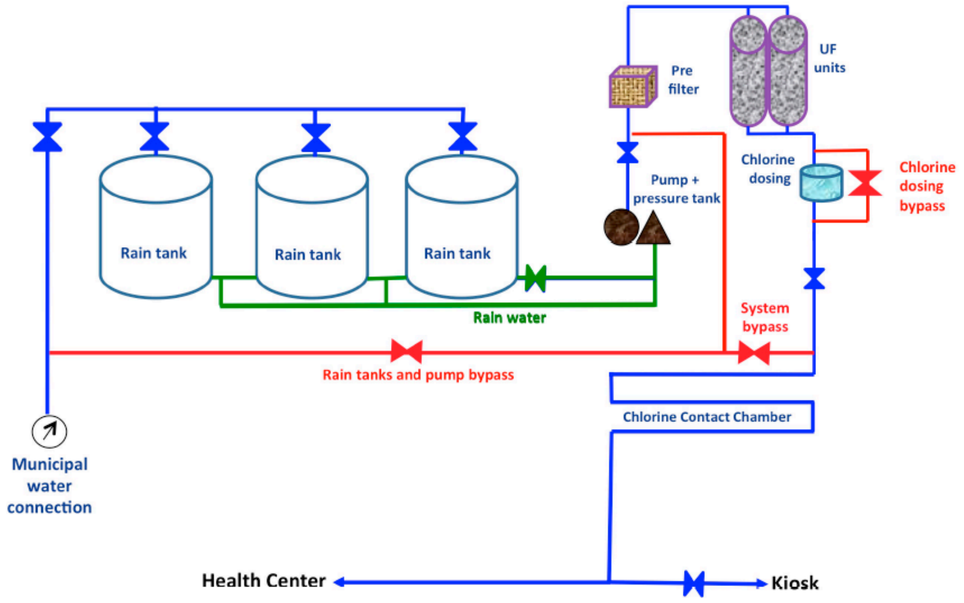

Figure S1. Schematic diagram of water treatment system at health center "C".

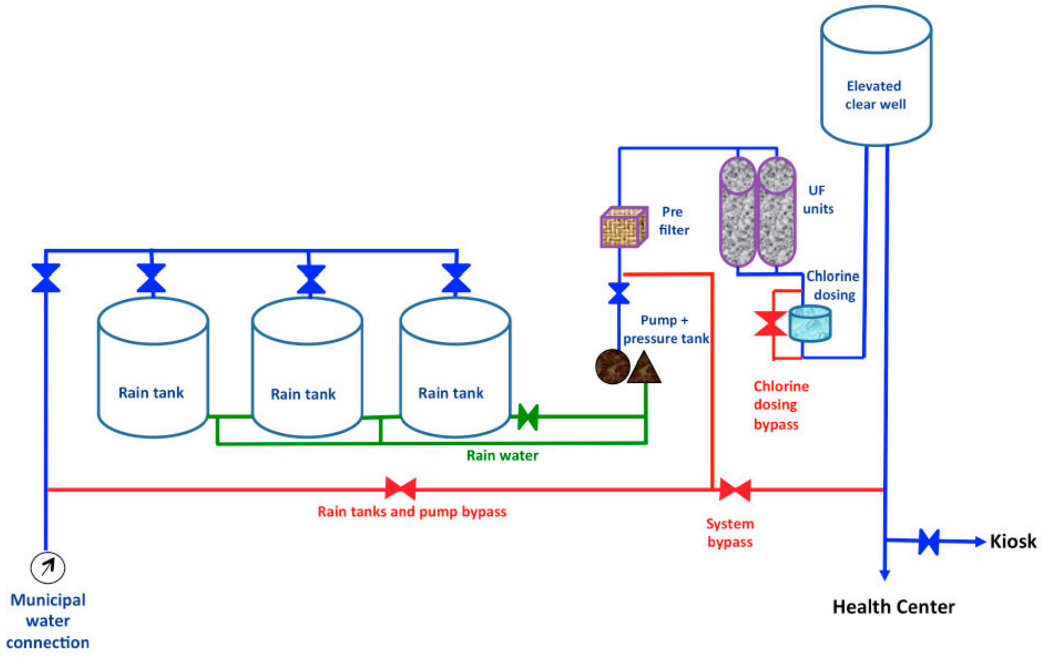

Figure S2. Schematic diagram of water treatment system at health centers "B" and "J".

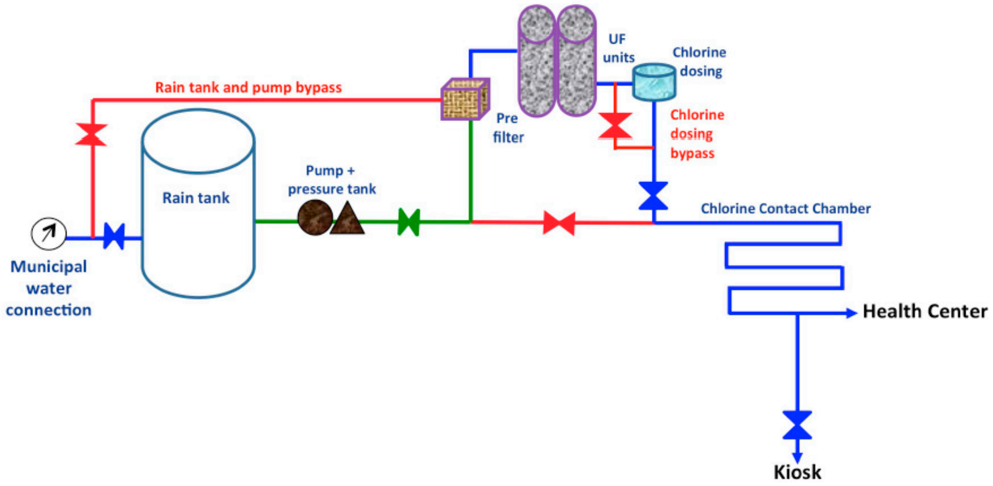

Figure S3. Schematic diagram of water treatment system at health centers "E", "F" and "G".

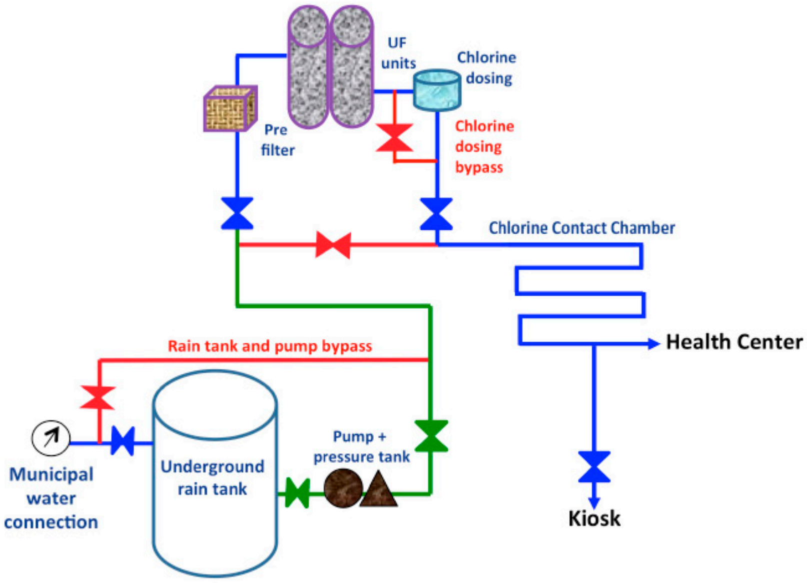

Figure S4. Schematic diagram of water treatment system at health centers "A" and "I".

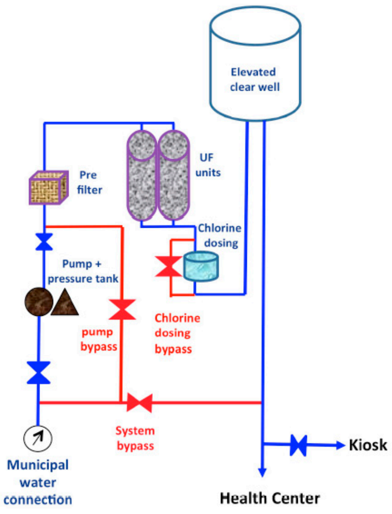

Figure S5. Schematic diagram of water treatment system at health centers "D" and "H".

Table S1. Quality of water from samples collected immediately following the water treatment systems when fully operational and during treatment interruptions.

Water quality indicator	WTS Fully Operational n (%)	Treatment Interruption * n (%)
Number of samples	40	7
E. coli (MPN **/100mL)		
<1	40 (100)	7 (100)
1–10	0 (0)	0 (0)
>10	0 (0)	0 (0)
Total Coliforms (MPN **/100mL)		
<1	40 (100)	6 (86)
1–10	0 (0)	1 (14)
>10	0 (0)	0 (0)
Free chlorine residual (mg/L) ***		
Mean	0.43	0.02
Median	0.02	0.02
Range	<0.02–2.20	<0.02–0.03
Total chlorine residual (mg/L) ***		
Mean	0.43	0.02
Median	0.1	0.06
Range	<0.02–2.20	0.03–0.07
Turbidity (NTU)		
Mean	0.59	0.89
Median	0.55	0.64
Range	0.01–1.29	0.22–2.68

* Treatment interruption indicates periods when the mechanisms for ensuring safe water at the point of use, such the chlorine dosing mechanism, were compromised. ** Most Probable Number. *** Limits of detection for free and total chlorine residual were 0.02 to 2.20 mg/L.

© 2015 by the authors; licensee MDPI, Basel, Switzerland. This article is an open access article distributed under the terms and conditions of the Creative Commons Attribution license (http://creativecommons.org/licenses/by/4.0/).
